# The effectiveness of the use of augmented reality in anatomy education: a systematic review and meta-analysis

**DOI:** 10.1038/s41598-021-94721-4

**Published:** 2021-07-27

**Authors:** Kerem A. Bölek, Guido De Jong, Dylan Henssen

**Affiliations:** 1 Department of Medical Imaging, Radboud University Medical Center, Geert Grooteplein Noord 21, 6525 EZ Nijmegen, The Netherlands; 2 Donders Institute for Brain, Cognition and Behavior, Radboud University Medical Center, Nijmegen, The Netherlands; 3 Radboudumc 3D Lab, Radboud University Medical Center, Nijmegen, The Netherlands

**Keywords:** Anatomy, Software, Computer science

## Abstract

The use of Augmented Reality (AR) in anatomical education has been promoted by numerous authors. Next to financial and ethical advantages, AR has been described to decrease cognitive load while increasing student motivation and engagement. Despite these advantages, the effects of AR on learning outcome varies in different studies and an overview and aggregated outcome on learning anatomy is lacking. Therefore, a meta-analysis on the effect of AR vs. traditional anatomical teaching methods on learning outcome was performed. Systematic database searches were conducted by two independent investigators using predefined inclusion and exclusion criteria. This yielded five papers for meta-analysis totaling 508 participants; 240 participants in the AR-groups and 268 participants in the control groups. (306 females/202 males). Meta-analysis showed no significant difference in anatomic test scores between the AR group and the control group (− 0.765 percentage-points (%-points); P = 0.732). Sub analysis on the use of AR vs. the use of traditional 2D teaching methods showed a significant disadvantage when using AR (− 5.685%-points; P = 0.024). Meta-regression analysis showed no significant co-relation between mean difference in test results and spatial abilities (as assessed by the mental rotations test scores). Student motivation and/or engagement could not be included since studies used different assessment tools. This meta-analysis showed that insufficient evidence is present to conclude AR significantly impacts learning outcome and that outcomes are significantly impacted by students’ spatial abilities. However, only few papers were suitable for meta-analysis, indicating that there is a need for more well-designed, randomized-controlled trials on AR in anatomy education research.

## Introduction

Anatomy education has historically been facilitated by cadavers, anatomical models and drawings in anatomical atlases^1^. In line with this, the anatomical assessment is based on the ability to recall spatial relationships between structures, both in two-dimensions (2D) and three-dimensions (3D)^2^. However, with an increasingly cramped curriculum for medical students, anatomy educator have been searching for engaging and interactive teaching methods based on state-of-the-art technologies^3^. Augmented reality (AR) concerns such a new technology which is believed to hold great potential for anatomy education^4,5^. AR has been defined as a technique that allows the user to superimpose virtual objects onto physical objects in real space and allows individuals to interact with both simultaneously. An essential difference with virtual reality concerns that with AR, the user is not completely immersed in a digital environment, which enables the user to combine digital input and real world objects^6^. With regard to the use of AR in anatomy education, AR can offer a highly realistic situated learning experience which is supportive to complex medical learning situations^7^. An important advantage of AR over physical models and cross-sections in learning anatomy, is that AR offers the opportunity to study the anatomy of a structure thoroughly by virtually disassembling and reassembling anatomical parts. A possible disadvantage of AR concerns the absence of tactile feedback^4^. It has been reported that, on a meta-level, three-dimensional visualization technologies yielded significant better results with regard to acquisition of spatial knowledge as compared to other teaching methods (i.e., dissection, cross-sections and 2D images)^8^.

Although the research concerning the implementation of AR in anatomical education is relatively limited, there are promising results regarding the teaching potential of AR^5,9^. Especially with regard to students’ motivation to study anatomy, various favorable reports have been published over the years^10–12^. The effects of AR on anatomy learning have also been investigated by various authors^13,14^. However, such studies are sparse and more evidence on a meta-study level is needed to investigate whether AR could effectively replace or supplement other anatomy teaching methods. Recently, three systematic reviews on the use of AR in anatomy learning were published^15–17^. However, two of the systematic reviews do not analyze pooled data^16,17^. In addition, the study of Moro et al., although published recently, does not only review the evidence on the use of AR in anatomy education, but also included study which investigated the use of AR in physiology education. Thereby, an unalloyed meta-analysis on the use of AR in anatomy education remains lacking in the recent scientific literature. This study therefore aimed to assess the effectiveness of AR in anatomy education. For that reason, we performed a systematic literature review and meta-analyzed the available quantitative evidence on the impact of AR on learning outcomes in anatomy education.

## Materials and methods

### Search strategy and data inclusion

The present study focuses on the effectiveness of learning anatomy by students by use of AR and was conducted following the Preferred Reporting Items for Systematic Reviews and Meta-Analyses (PRISMA) guidelines^18^. To assess a wide number of eligible papers, exploratory searches were carried out to assess suitability of literature databases (e.g. ACM digital library, Education Resources Information Center (ERIC), PubMed, Sciencedirect, PsychINFO, Google Scholar). Exploratory searches were conducted until March 2020. Thereafter, an independent librarian was consulted to help the researchers to identify the most suitable database to obtain literature from and to construct suitable search strategies. Various databases (i.e., Pubmed, Embase, ERIC The Cochrane Library, Google Scholar) were then searched systematically. Searches were conducted until January 2021. Search strings per database are provided in the Supplementary files. There was no restriction in the search strategy with regard to publication date. Additionally, the authors (K.B. and D.H.) hand-searched the reference lists of relevant systematic reviews and included papers. One of the authors (D.H.) contacted corresponding authors of papers when data was missing or when clarification was needed. Selection of relevant articles was carried out by two researchers independently (K.B. and D.H.). The papers eligible for inclusion were original research reports of a comparative study in which the research aim was to investigate the effects of AR on post-intervention anatomic knowledge in university-level human anatomical education. These effects needed to be evaluated by any other form of anatomical education (e.g., dissection, atlas-based learning etc.). Case reports, editorial commentaries, systematic or narrative reviews and articles that did not meet the inclusion criteria were excluded.

**Figure 1:**
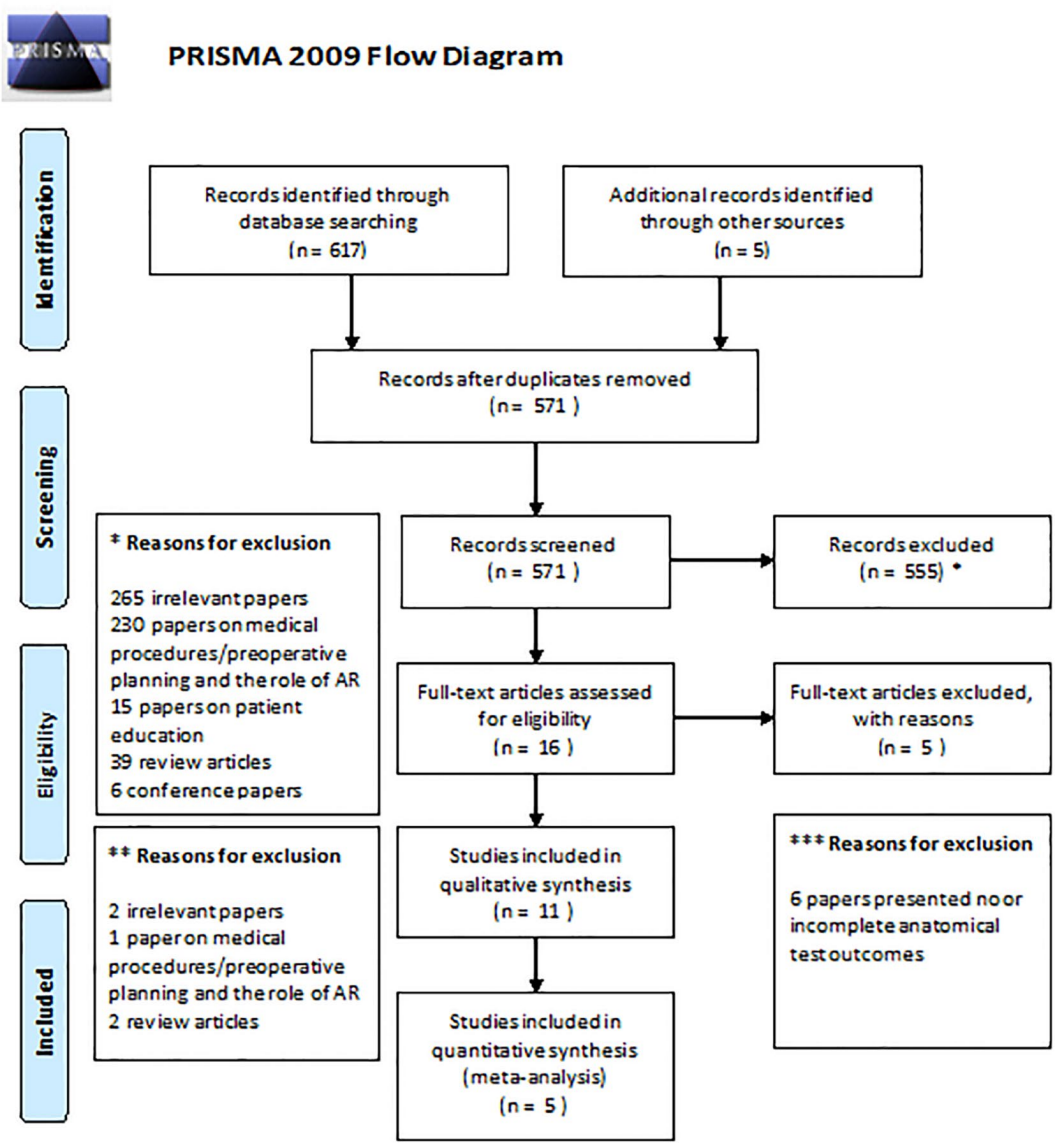
PRISMA flow diagram for the systematic review detailing the database searches, the number of abstracts screened and the full texts retrieved.

The first round of assessment of the obtained papers concerned screening title and/or abstract. The second round of assessment comprised full-text assessment and included whether these articles met the aforementioned inclusion criteria to be included. When in disagreement, a third investigator (G.d.J.) was contacted to make the final decision. The PRISMA flow diagram can be appreciated in Fig. 1.

After inclusion, data were extracted from the individual papers using a data extraction sheet by two authors independently (K.B. and D.H.). These data included: (1) type of AR used in the study, (2) type of anatomical education in the control group, (3) number of participants, (4) characteristics of the included participants (i.e., sex, age, study direction), (5) type of anatomical test, (6) mean post-intervention anatomic test scores for the experimental (AR) group, (7) mean post-intervention anatomic test scores for the control group and (8) Mental Rotations Test (MRT) scores in percentages of each included group as this test assesses the spatial abilities of participants. When the design of the study was a multiple group comparison study, each individual group that was not using AR was considered a separate control group. All control groups were then included for the meta-analysis.

### Quality assessment and risk of bias

The quality of the evidence of the studies was graded by two authors independently (K.B. and D.H.) according to the GRADE approach guidelines defined by The Cochrane Collaboration’s Handbook^19^. Additionally, risk of bias was assessed by two authors independently (K.B. and D.H.). Discrepancies were resolved by discussion or reference to a third author (G.d.J.). Risks of biases which were assessed included: selection bias (criteria 1, 2, 9), performance bias (criteria 3, 4, 10, 11), attrition bias (criteria 6, 7), detection (or measurement) bias (criteria 5, 12) and reporting bias (criterion 8). Also, the Kirkpatrick’s model of change of knowledge was assessed for each paper as well. This model evaluates the learning outcomes and classifies these in four levels: 1) reaction; 2A) learning (change in attitude); 2B) learning (modification of knowledge or skills; 3) behavior (change in behavior); 4A) results (change in the system/organizational practice); and 4B) results (improvement in learner performance)^20,21^. Each potential source of bias was graded as low, high, or unclear. Assessing the risk of bias was performed by the criteria presented in Table 1 following standardized instructions^19^. In addition, the second version of the Cochrane risk-of-bias tool for randomized trials (RoB 2) was used to assess the risk of bias in the included randomized trials (Table 1).

**Table 1:** Quality assessment of the evidence provided by the individual papers.

Study	Internal validity												Score	Quality	Level in Kirkpatrick’s model
	1	2	3	4	5	6	7	8	9	10	11	12			
Moro et al. 2017	+	−	−	−	−	−	+	+	+	+	+	+	60%	Moderate	2B
Barmaki et al. 2019	+	−	−	−	−	+	+	+	+	+	+	−	60%	Moderate	2A, 2B
Bork et al. 2019	−	−	−	−	−	+ *	+	+	+	+	+	+	60%	Moderate	2A, 2B
Henssen et al. 2019	+ *	−	−	−	+	+	+	+	+	+	+	+	75%	Moderate	2A, 2B
Bogomolova et al. 2020	+	−	−	−	−	+	+	+	+	+	+	+	75%	Moderate	2A, 2B

1. Was the method of randomization adequate?

2. Was the allocation concealed?

3. Was the participant blinded to the intervention?

4. Was the teacher blinded to the intervention?

5. Was the outcome assessor blinded to the intervention?

6. Was the dropout rate described and acceptable?

7. Were all randomized participants analyzed in the group to which they were allocated?

8. Are reports of the study free of suggestion of selective outcome reporting?

9. Were the groups similar at baseline regarding the most important prognostic indicators?

10. Were co-interventions avoided or similar?

11. Was the compliance acceptable in all groups?

12. Was the timing of the outcome assessment similar in all groups?

+ , criterion achieved; –, criterion not achieved; ∗ , assessors initially disagreed.

High: > 75% of the criteria have been fulfilled [≥ 10/12]. Where they have not been fulfilled the conclusions of the study or review are thought very unlikely to have been altered.

Moderate: 50–75% of the criteria have been fulfilled [6–9/12]. Those criteria that have not been fulfilled or not adequately described are thought unlikely to have altered the conclusions.

Low: Less than 50% of the checklist criteria were fulfilled [< 6/12]. The conclusions of the study are thought likely or very likely to alter had those criteria been fulfilled^54–63^.

Levels of change of knowledge according to the model of Kirkpatrick: (1) reaction; (2A) learning (change in attitude); (2B) learning (modification of knowledge or skills; (3) behavior (change in behavior); (4A) results (change in the system/organizational practice); and (4B) results (improvement in learner performance)^20,21^.

Printed below is the overview of the quality assessment as assessed by the second version of the Cochrane risk-of-bias tool for randomized trials (RoB 2).

### Statistical analysis

The statistical package SPSS Statistics, version 25 (IBM Corp., Armonk, NY) was used for descriptive statistical analyses of the aggregated data. Descriptive statistical analyses were represented as mean with ± standard deviation (± SD). Meta-analysis with continuous random-effects was carried out by use of the visual front-end for the R-package (https://www.r-project.org; Metafor)^22^: OpenMeta[Analyst] software (MetaAnalyst, Tufts Medical Center (Wallace et al., 2012)). A forest-plot was created to graphically display the estimated differences in pre-intervention and post-intervention test results from the included studies, along with the overall results. Cohen’s d metric was used to assess the effect size. In addition, OpenMeta[Analyst] was used to assess heterogeneity. Heterogeneity in meta-analyses refers to the variation in outcomes between included studies. To measure heterogeneity, Cochran's Q was calculated as the weighted sum of squared differences between individual study effects and the pooled effect across studies. To improve interpretation, the heterogeneity index (I_2_), defined as the proportion of total variability explained by heterogeneity and refers to the percentage of variation across studies, was introduced^23^. I_2_ is independent from the number of studies included in the meta-analysis. Therefore, I_2_ highlights the inconsistency across studies and ranges from 0% (i.e., no heterogeneity) to 100% (i.e., the highest heterogeneity).

### Ethical approval

Ethical approval was not applicable for conducting this systematic review and meta-analysis.

## Results

Systematic searching and systematic assessment of the retrieved papers resulted in the inclusion of five papers in which AR was compared with another form of anatomical learning, as shown in Fig. 1^13,14,24–26^. The assessment for the risk of bias and the level of change of knowledge according to the model of Kirkpatrick is summarized in Table 1. See Table 2 for more information on the participants in the included studies. All papers showed to be of moderate quality with minimal risks of bias.

**Table 2:** Specifications of the included studies and characteristics of the included participants.

Study	Anatomy learning task	Type of AR feature	Comparison	Subjects in each group (n)	Mean age (years) (± SD)	Gender (F/M)	Study (MED/ BMS)	Mean test-score in the different groups (%) (± SD)	Mean difference in test-scores (percentage points)	Lower bound – Upper bound (percentage points)
Moro et al. 2017	Studying anatomy of the bones of the skull	(1) Tablet-based AR application presenting 3D model of the bones of the skull	(2) Headset-based VR application (3) Tablet-based non-AR three dimensional model	(1) 17 (2) 20 (3) 22	(1) 19.5 ± 2.3 (2) 20.2 ± 3.5 (3) 22.2 ± 8.0	(1) 7/10 (2) 12/8 (3) 12/10	N/A	(1) 62.5 ± 17.1* (2) 64.5 ± 18.5* (3) 66.5 ± 18.5*	(1–2) − 2.0% (1–3) − 4.0%	(1–2) − 13.5 to 9.5% (1–3) − 15.1 to 7.2%
Barmaki et al. 2019	Body painting of musculoskeletal anatomy of the upper and lower limb	(1) REFLECT; virtual mirror with augmented anatomical over-projection	(2) No REFLECT; virtual mirror without augmented anatomical over-projection	(1) 164 (2) 124	Total: 19.8 ± 2.0	Total: 178/110	N/A	(1) 43.0 ± 28.4 (2) 39.2 ± 28.8	(1–2) 3.8%	(1–2) − 2.9 to 10.5%
Bork et al. 2019	Studying gross anatomy of body parts (pelvis, shoulder, chest, abdomen, and extremities)	(1) MagicMirror; virtual mirror with augmented anatomical over-projection	(2) Anatomage; a virtual dissection table (3) Traditional, 2D anatomical atlases	(1) 24 (2) 24 (3) 24	Total: 21.4 ± 3.4	Total: 49/23	N/A	(1) 56.0 ± 14.1 (2) 55.2 ± 11.0 (3) 59.1 ± 16.9	(1–2) 0.8% (1–3) − 3.1%	(1–2) − 6.3 to 8.0% (1–3) − 11.9 to 5.7%
Henssen et al. 2019	Studying neuroanatomy	(1) GreyMapp; tablet-based AR application presenting a 3D model of the human brain	(2) Cross-sections of the human brain	(1) 15 (2) 16	(1) 19.3 ± 2.3 (2) 19.1 ± 0.8	(1) 6/9 (2) 6/10	(1) 13/2 (2) 10/6	(1) 50.0 ± 10.2 (2) 60.6 ± 12.4	(1–2) − 10.6%	(1–2) − 18.6 to − 2.6%
Bogomolova et al. 2020	Studying lower limb anatomy	(1) Headset-based AR application	(2) Non-AR 3D desktop model (3) Traditional, 2D anatomical atlases	(1) 20 (2) 20 (3) 18	(1) 18.5 ± 0.8 (2) 18.7 ± 1.0 (3) 18.7 ± 0.7	(1) 12/8 (2) 13/6 (3) 11/7	(1) 17/3 (2) 16/4 (3) 14/4	(1) 47.8 ± 9.8 (2) 38.5 ± 14.3 (3) 50.9 ± 13.8	(1–2) 9.3% (1–3) − 3.1%	(1–2) 1.7–16.9% (1–3) − 10.8 to 4.6%

*AR* augmented reality, *BMS* biomedical sciences, *F* female, *M* male, *MED* medicine, *N/A* not available, *VR* virtual reality.

*Standard deviations were derived from Boxplot analysis.

### Study characteristics

The initial search yielded 430 results found in different databases of which 23 were duplicates and removed. Evaluating the title and abstract, 43 records were chosen to be screened. Of these, 12 papers were eligible for the qualitative synthesis. After evaluating full text, 12 papers were found to match our inclusion criteria, of which 7 proved to be irrelevant to our aim. The 5 remaining papers met the inclusion criteria. However some of the required outcomes, such as student motivation was not reported in all of the papers. The PRISMA flowchart shows the details and the search strategy can be found in the Supplementary files. The assessment of the risk of bias was done according to the model of Kirkpatrick and is summarized in Table 1. The studies were synthesized by identifying the similar key themes and statements in these papers and then by independent reviews and later consensus building reclassifying these similarities and gathering conclusions from them following the PICO framework.

### Participant variation

The total number of participant was 569, of which 306 were female. Participants originated from several countries, namely Australia, United States, Germany and the Netherlands. Undergraduates studying anatomy were sought out. The five studies have similar age groups, with the clear outlier of one paper’s third group^26^. The means range from 18.5 to 22.5 years of age. Three studies reported the ratio of included biomedical students to medical students^14,24,25^, which can be seen in Table 2. The groups show similarities in age, future academic aims and MRT scores. The effect of MRT scores has been examined in three papers^13,24,25^. MRT scores showed to have an significant impact on the pre and posttest scores. Bork et al. showed that participants with low MRT scores using AR had higher scores compared to control, which was in accordance with the findings of Bogomolova et al., 2020.

### Intervention heterogeneity

The AR interventions show differences in their approach to AR. Henssen et al., 2019 and Moro et al., 2017 shows a practical tablet based 3D model, while two studies opted for virtual mirrors with AR capabilities, called REFLECT^13,14^. This mirror possess the ability to virtually project musculature on a subject. A headset-based AR application has been used in one study^24^. All these interventions conform to the definition of AR. However, the differences should be noted in the form of AR and the implications, such as the adverse events reported by Moro et al., 2017. These showed that AR users experienced more general discomfort in their use compared to tablet users^26^. Henssen et al., 2019 reported that students needed to get used to the device, causing some discomfort. Magic Mirror was claimed to be tiring to use after long learning sessions, according to three participants from Bork et al. 2019 while no such feedback was given in Barmaki et al., 2019. Moreover, no adverse effects were reported by Bogomolova et al., 2020.

### Controls

Traditional teaching methods have been used, such as cross-sections and anatomical atlases, by three studies^13,24,25^. Two of these studies used a virtual dissection table and a non-AR 3D desktop model respectively, while the latter had cross-sections as control. In the study of Barmaki et al. 2019 the virtual mirror without superimposing AR features functioned as control. Moro et al., 2017 compared AR to a VR headset and a conventional tablet based 3D model.

### The effects on learning

The primary outcome measure was the effectiveness on learning, measured with the difference in pre- and posttest scores. The tests consisted of multiple choice questions in all of the studies, where some studies opted to supplement the tests with open ended questions, regarding the chosen anatomical structures. Little to no significant difference was found in the effectiveness on learning anatomy when looking at test scores. Notwithstanding, Bork et al. reported that the AR group did score significantly higher than the virtual dissection table (Anatomage) group. However, no difference between the conventional atlas group and the AR group was found^13^. Conversely, Barmaki and colleagues found REFLECT users did score significantly higher than their virtual mirror controls^14^. MRT scores showed to be of importance as several studies found that students with lower MRT scores learned more with the 3D AR models than with conventional materials.

### Secondary outcomes

In the study of Moro et al., 2017 adverse effects were reported for the VR study tool, which caused students to experience nausea, headaches and dizziness. No such symptoms and problems plagued the use of their AR tool. Discomfort was also experienced by students using GreyMapp, as they reported trouble with getting used to operating the application. In combination with taking notes during the lesson, some students assumed uncomfortable positions to multitask. This problem was easily solved by creating a bigger tablet interface. In the REFLECT study, it was reported that time on task increased significantly. In addition, students engagement was significantly higher in the AR group, causing the longer time on task.

Henssen et al. reportedly did not find an increase in motivation when comparing the AR group to the conventional group. However, focus group interviews showed that students did find the concept novel and interesting. Additionally, some students expressed their disappointment with not being able to work with the program^25^. Engagement was gauged differently in the study of Barmaki et al., 2019, where they measured time on task has been suggested as an important marker for knowledge retention and student engagement. The time on task was significantly higher in the AR group, compared to controls (P = 0.01). Finally, a significant difference was found by Bogomolova et al. in the enjoyment during learning between 2D anatomical models and the AR intervention (P = 0.003)^24^. Table 2 summarizes the outcomes.

### Meta-analysis

**Figure 2:**
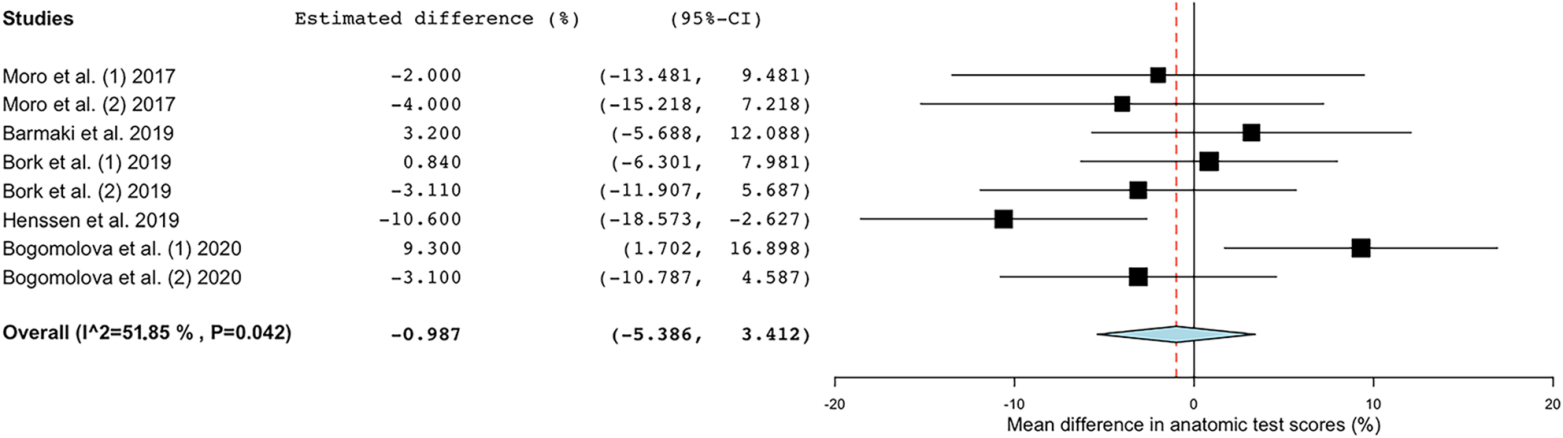
Forest plot showing the estimated mean difference in anatomic test scores (%) from the different included studies investigating AR as compared with other forms of anatomical education. *AR* augmented reality, *95%-CI* 95%-confidence interval.

**Figure 3:**
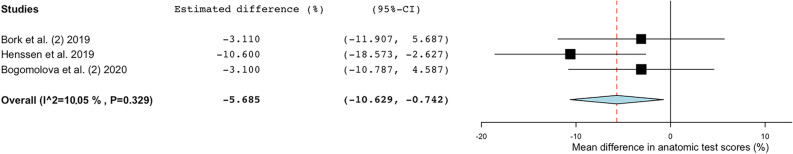
Forest plot showing the estimated mean difference in anatomic test scores (%) from the included studies addressing AR vs. 2D forms of anatomical education (i.e., traditional anatomical atlases, radiological data). *AR*  augmented reality, *95%-CI* 95%-confidence Interval.

**Figure 4:**
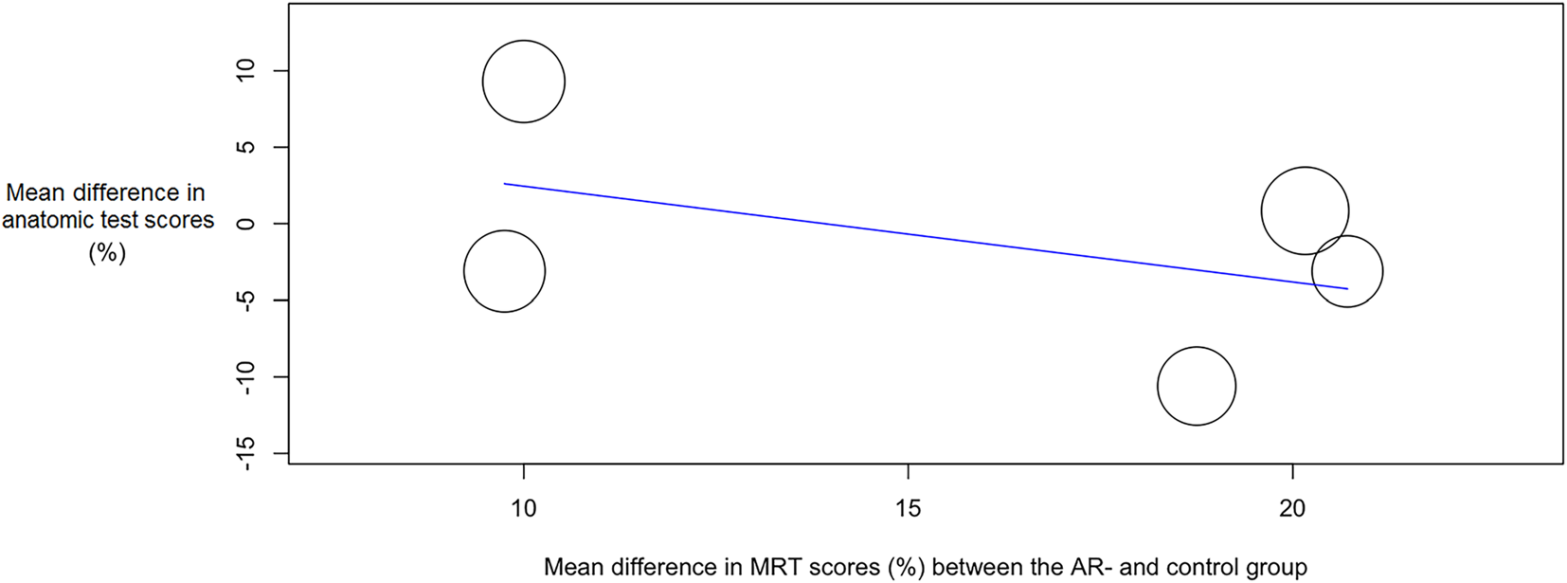
Bubble plot with fitted meta-regression line of mean difference in anatomic test scores (%) and spatial ability. Included are the studies addressing AR vs. 2D forms of anatomical education (i.e., traditional anatomical atlases, radiological data). *AR* augmented reality, *MRT* mental rotation test.

Meta-analysis showed a substantial heterogeneity in the included papers (Tau^2^ = 21.301; Q = 15.493; df = 7; I_2_ = 54.82%; P = 0.030). Based on the mean differences in anatomic test scores (percentage-points; %-points) between the AR groups and the control groups, a difference of -0.765%-points was estimated (P = 0.732; Cohen’s d = -0.35). This indicated that there was no significant advantage or disadvantage when learning anatomy with AR (Table 2; Fig. 2). Sub analysis was carried out on studies using 2D anatomy teaching methods as a comparison to AR-based learning^13,24,25^. This sub analysis showed significant lower mean anatomic test scores for the AR-groups (P = 0.024) in studies which showed a low interstudy heterogeneity (Tau^2^ = 1.927; Q = 2.224; df = 2; I_2_ = 10.05%; P = 0.329), as seen in Fig. 3. In order to observe whether outcomes of the different groups (AR vs. control groups) are impacted by spatial abilities of the participants, a meta-regression analysis was performed for the studies that (1) compared AR-features with 2D anatomy teaching methods and (2) used a MRT to assess spatial ability^13,24,25^. Meta-regression showed no significant relation between mean difference in anatomic test results (%) and mean difference in MRT scores (%) between the AR- and control-groups (Omnibus P = 0.229), which can be appreciated in Fig. 4.

## Discussion

Although cadavers are most commonly used for teaching.

Although the use of cadavers and/or prosections form the cornerstone of anatomical education for medical and biomedical sciences students, various limitations constrain their use (for a recent overview, see^27^). Therefore, various other teaching methods are merited, including AR. AR is explicitly useful in anatomical education as it presents the first consumer-grade technology that can depict realistic 3D models and concepts to students, which, at the same time, can be directed by a teacher^28^. However, the present meta-analysis showed that AR yields no significant learning benefits when compared to other forms of anatomical education. Moreover, a significant lower anatomic test score was observed when comparing the results from the AR-groups to groups that used 2D anatomical learning methods (e.g., traditional anatomical atlases and cross-sections). The results from the present meta-analysis partially conflict with the results from the meta-analysis of Yammine and Violato (2015) in which it was found that three dimensional visualization techniques (1) resulted in higher factual knowledge, (2) yielded significant better resulted in spatial knowledge acquisition, and (3) produced significant increase in user satisfaction and in learners’ perception of the effectiveness of the learning tool^8^. However, these three dimensional visualization techniques included various 3D images, annotated radiological data and VR simulators and that did not include AR features. On the use of AR in anatomical education, two other recent meta-analyses have been published. The publication of Moro et al. (2020), although also integrating VR methods and non-anatomical education purposes (e.g., physiology education), demonstrated that VR and AR can be used as delivery methods in medical education, without any adverse effects on student performance^29^. Although not supported by their analyses, Moro et al. also expressed that there is a chance that the use of these technologies may have a positive impact on students spatial understanding and 3D comprehension of anatomical structures^29^. A second meta-analysis, however focusing on VR, showed that VR may act as an efficient way to improve the learners’ level of anatomy knowledge^30^. The present meta-analysis partially contradicts the conclusions of the other studies, showing that AR can indeed worsen the learners’ performance when compared to 2D anatomy teaching methods. An explanation for these different outcomes can be explained by the fact that the present study maintained strict inclusion criteria and thereby only focused on the effects of AR in anatomy education. On the one hand, this could have purified the results, whereas on the other hand, this could cause an overestimation of the effects related to a limited sample size.

### Impact on the literature and anatomy education practices

This literature review and meta-analysis provided recommendations which should be further investigated in the near future. These recommendations include the investigation of students’ engagement, motivation and cognitive load when working with AR. Furthermore, it remains unelucidated whether different AR tools elicit different learning outcomes and/or student behavior. In daily practice, anatomy education is facilitated by cadavers, models and drawings (Mclachlan et al., 2006, Kurt et al., 2013). However, the use of cadavers is known to have practical and ethical drawbacks^31^. The lack of other teaching tools in some countries and increased use of technological methods of teaching merit a more up to date, alternative method. Based on this meta-analysis, the authors concluded that AR could serve as such a beneficial education tool.

### Spatial ability, cognitive load and the use of AR

One of the co-variates in most studies investigating AR concerns spatial ability. Most studies use the MRT to assess spatial ability of participants. The MRT assesses mental visualization and mental rotation, which are considered the main components of visual-spatial abilities. The MRT concerns a 24-item psychometric questionnaire designed in 1971^32^ and previously validated by Vandenberg and Kuse (1978)^33^. The findings of three of the included studies that used MRT^13,24,25^ showed that an aptitude–treatment interaction caused by visual-spatial abilities needs to be considered when reviewing evidence of AR in anatomical learning. However, no significant correlation was found between the mean difference in anatomic test scores and the MRT scores of the different groups in this meta-analysis. This could be due to the fact that only limited data was available. On the contrary, previous studies which focused on spatial ability and the use of 3D visualization methods found that significant differences in pre-intervention spatial ability confounded the study results^34–37^. Still, various reports have shown that cognitive load decreases when students study anatomy by use of AR^25,38^. This could, however, not be incorporated into this meta-analysis as most of the included papers did not provide this information.

### Motivation and student engagement

Numerous studies reported improvements in the learners’ motivation after implementation of AR in different fields of education^39–42^. Literature has suggested that AR would be attractive to students, increasing their motivation to learn anatomy^43–45^. Several studies investigated various forms of student motivation with regard to learning anatomy. For example, Allen et al. (2016) reported that students felt confident that learning with 3D models, including AR 3D models, could help them to understand anatomical concepts. Also, the majority of the respondents would encourage the development of similar learning sources^46^. Kucuk et al. distilled from interviewing students that more permanent learning was achieved in a shorter time by using AR^38^. Such permanent learning, however, remains rather understudied in research on AR in anatomy education^47^. Another report by our group showed that students feel motivated to study neuroanatomy by use of AR, although men and women and students from different study directions have different attitudes towards learning with AR. As well, students expressed that they felt AR was especially beneficial to study structures that cannot be visualized properly by use of prosected cadavers (i.e., the subcortical structures of the brain)^48^. Although most of the included studies in the present meta-analysis included motivation as a (secondary) outcome measure^13,14,25,26^, there is still no validated method to measure students’ motivation for learning anatomy^49^. Therefore, this could not be included into this meta-analysis. Future research elucidating methods of gathering data on student motivation will therefore provide valuable insights. In addition, the novelty effect, which is defined as “a person’s subjective first response to (using) a technological innovation”, plays an important role in the studies that used AR as an anatomical teaching method^50^. Previous studies noted that as the novelty effect wears off, users discontinue their use of new technologies, indicating a loss of interest and motivation^50,51^. This could partially be explained by the law of diminishing returns, as novel technologies create inherent interest, which tapers off after students get familiarized with their new environments^52^.

### Different types of AR interventions and effectiveness

Within this review, different types of AR were investigated ranging from practical tablet based 3D models, virtual mirrors with AR capabilities and headset-based AR applications. However, much debate still remains with regard to the most optimal implementation of different AR techniques in the correct period of a course. For example, in the study of Kügelmann et al. (2018), students were presented with a mirror AR system by which they could explore radiological images in several anatomical intersection planes in an attempt to increase motivation to study anatomy. The participants were asked to fill in questionnaires with regard to the levels of motivation and teaching potentials of the studied AR system. The participants declared that the benefits of AR were enhanced as time passed during the course^53^. Unfortunately, the lack of a statistical analyses partially limits the interpretation of this finding. Furthermore, whether this is also the case for the use of AR when studying three dimensional anatomy remains unclear. With regard to the most effective AR technique in anatomy education, much remains unknown. For example, Chytas et al. (2020) demonstrated in their review that AR technology has a remarkable teaching potential with encouraging learning outcomes. However, it was noted that these papers generally compared tablet-based AR features which could enhance anatomical images and/or text with the anatomical atlases and/or textbooks themselves. In the minority of papers, three-dimensional AR applications were studied. In addition, comparisons with cadavers remains a relatively understudied field^5^. Based on the findings of the present study, we could carefully suggest that virtual mirrors with AR capabilities could help students to learn anatomical relations in the most effective way. These mirrors allow users to see a reflection of themselves with virtual information superimposed on a large display which acts as a digital representation of a mirror. Thereby, they can interact with otherwise invisible anatomical structures in real-time whilst benefiting from the anatomical context of their own bodies.

### Strengths and limitations

One of the strengths of the present meta-analysis concerns the systematic search for available literature and the independent consideration of each paper prior to inclusion and the independent assessment of the risk of bias, level of change in education as defined by Kirkpatrick and the results. A limitation of the present meta-analysis concerns the heterogeneity of the included papers, especially with regard to the different teaching methods in the intervention group (e.g., Magic Mirror AR, tablet-based AR model). However, to the authors knowledge, no studies exists which show that different AR modalities are cognitively processed in a different fashion. Also, no studies were found which investigated the cognitive load with regard to the used AR teaching methodology. Therefore, we cannot determine the effects of these different AR modalities on a meta-level with regard to percentage-point differences in anatomy tests. Another limitation of the current meta-analysis concerns the relatively limited amount of papers included. However, a strengths concerns the strict inclusion and exclusion criteria which resulted in a meta-analysis which focuses on AR technologies in anatomy education only. In addition, testing of anatomical knowledge was performed by using a combination of multiple-choice questions, matching questions and open-ended questions. One of the strengths of the meta-analysis is caused by the consequent use of a validated MRT^32,33^ to assess spatial ability in the included studies. A limitation, on the other hand, is caused by the lack of validated tools to evaluate students’ engagement, motivation and cognitive load.

## Conclusions

This meta-analysis suggested that AR has no significant beneficial or disadvantageous effects on students’ learning anatomy when compared with various traditional educational tools. For that reason, we concluded that AR could be a viable addition to traditional anatomy education in an increasingly technological world.

## Supplementary Information

Supplementary Information.
