# Supplementary material for: The effectiveness of the use of augmented reality in anatomy education: a systematic review and meta-analysis
Source: Sci Rep. 2021 Jul 27;11:15292. doi: 10.1038/s41598-021-94721-4 (PMC8316386; doi:10.1038/s41598-021-94721-4)
Supplement: Supplementary file 1 — Supplementary Information. [file 41598_2021_94721_MOESM1_ESM.docx]

Supplementary files

The effectiveness of the use of augmented reality in anatomy education: a systematic review and meta-analysis

**Kerem A. Bölek^1^, Guido De Jong^3^, Dylan J.H.A. Henssen^1,2^***

^1^Department of Radiology, Nuclear Medicine and Anatomy, Radboud University Medical Center, Nijmegen, the Netherlands

^2^Donders Institute for Brain, Cognition and Behavior, Radboud University Medical Center, Nijmegen, the Netherlands

^3^Department of Neurosurgery, Radboud University Medical Center, Nijmegen, the Netherlands

Running title: Effectiveness of AR in (neuro)anatomy education

| **#** | **Search string** | **Number of hits** |
| --- | --- | --- |
| 1 | Augmented reality.m_titl. | 1201 |
| 2 | exp medical student/ or exp anatomy/ or exp education/ or exp medical education/ or exp teaching/ | 1508422 |
| 3 | Augmented reality.mp. | 2378 |
| 4 | (Augmented reality and Anatomy education).ti. | 1 |
| 5 | 1 and 2 | 258 |
| 6 | anatomy education.ti. | 232 |
| 7 | 3 and 6 | 3 |
| 8 | (Augmented reality and Anatomy education).af. | 15 |

**Embase**

Accessed until March 27^th^ 2020

**PubMed**

Use the table below to construct a systematic review search strategy for MEDLINE (PubMed).

Fill out row 1 with the [Mesh] terms for your population, combining them with OR. Fill out row 2 with the [tiab] terms for your population, combining them with OR.

Fill out row 4 with the [Mesh] terms for your intervention, combining them with OR. Fill out row 5 with the [tiab] terms for your intervention, combining them with OR.

Add additional rows as needed (if you are searching for outcome terms, for example).

Run each row in PubMed Advanced Search, and report the **# of results.**

| **#** | **Search strategy** | **# of results** |
| --- | --- | --- |
| 1 | (Anatomy[MeSH Terms]) AND Education[MeSH Subheading]) | 4397 |
| 2 | Anatomy[Title/Abstract] | 123976 |
| 3 | Anatomy[MeSH Terms] | 393902 |
| 4 | #2 OR #3 | 507746 |
| 5 | "Augmented Reality"[Mesh] | 58 |
| 6 | “Augmented Reality” [Title/Abstract] | 1880 |
| 7 | #5 OR #6 | 1889 |
| 8 | Educational measurement[MeSH Terms] | 146229 |
| 9 | #4 AND #7 | 14 |
| 10 | #2 AND #6 | 1 |
| 11 | #3 AND #5 | 147 |
| 12 | randomized controlled trial [pt] | 502961 |
| 13 | #1 AND # 7 AND #12 | 0 |
| 14 | #8 AND #9 | 0 |
| 15 | #9 AND #12 | 3 |
| 13 |  |  |

Accessed until March 27^th^ 2020

Developed by Kate Ghezzi-Kopel, Health Sciences & Evidence Synthesis Librarian,July 13, 2018

**ERIC**

| **#** | **Search string** | **Number of hits** |
| --- | --- | --- |
| 1 | “Anatomy” | 3209 |
| 2 | "Augmented Reality" OR "AR" | 1328 |
| 3 | 1 and 2 | 18 |

| **#** | **Search string** | **Number of hits** |
| --- | --- | --- |
| 1 | exp *Medical Education/) or exp *Anatomy/ | 4289 |
| 2 | ((exp *Technology Uses in Education/ or exp *Educational Technology/ or exp *Handheld Devices/ or exp *Computer Simulation/ or exp *Simulated Environment/ or exp *Educational Games/) | 14998 |
| 3 | 1 AND 2 | 169 |

Accessed until October 6^th^ 2020
